# Role of B cells as antigen presenting cells

**DOI:** 10.3389/fimmu.2022.954936

**Published:** 2022-09-08

**Authors:** Ichwaku Rastogi, Donghwan Jeon, Jena E. Moseman, Anusha Muralidhar, Hemanth K. Potluri, Douglas G. McNeel

**Affiliations:** University of Wisconsin Carbone Cancer Center, University of Wisconsin-Madison, Madison, WI, United States

**Keywords:** B cells, professional APC, antigen processing, antigen presentation, TIL-B, cellular therapies

## Abstract

B cells have been long studied for their role and function in the humoral immune system. Apart from generating antibodies and an antibody-mediated memory response against pathogens, B cells are also capable of generating cell-mediated immunity. It has been demonstrated by several groups that B cells can activate antigen-specific CD4 and CD8 T cells, and can have regulatory and cytotoxic effects. The function of B cells as professional antigen presenting cells (APCs) to activate T cells has been largely understudied. This, however, requires attention as several recent reports have demonstrated the importance of B cells within the tumor microenvironment, and B cells are increasingly being evaluated as cellular therapies. Antigen presentation through B cells can be through antigen-specific (B cell receptor (BCR) dependent) or antigen non-specific (BCR independent) mechanisms and can be modulated by a variety of intrinsic and external factors. This review will discuss the pathways and mechanisms by which B cells present antigens, and how B cells differ from other professional APCs.

## 1 Introduction

B cells, commonly considered as antibody factories, are best known for their contribution to humoral immunity. They were first identified and described by Max Cooper in the 1960s, when he demonstrated that an irradiated chicken completely loses its ability to generate antibodies after removal of the Bursa of Fabricus, the primary site for B cell development in birds (1). Ever since, researchers have continued to explore the biology of B cells, with a focus on their role in antibody production. In humans, B cells originate and develop in the bone marrow and undergo selection and maturation in secondary lymphoid organs, predominantly in the spleen (2). B cells can be identified by the presence of a B cell receptor (BCR) on their surface, which also defines the antigen specificity of the cell. Moreover, B cells also express both major histocompatibility complex (MHC) I and MHC II, and are equipped with all the machinery required for antigen uptake, processing, and presentation. Hence, they are also classified as professional APCs, like dendritic cells (DC), monocytes and macrophages.

Recent studies have demonstrated that B cells can affect cancer progression. The role of B cells in cancer is complex, with some reports demonstrating pro-tumorigenic behavior for B cells and others showing enhancement of anti-tumor responses. Studies in murine models, in particular, have shown that B cell knockout mice exhibit better tumor control, that B cells can cause increased angiogenesis, and that B cells can cause circulating immune complex deposition which favors carcinogenesis (3–5). Furthermore, an IL-10-producing subset of B cells, known as B regulatory cells (Bregs), has been identified in both murine and human cancers. Bregs have been shown to be capable of converting conventional CD4+ T cells to T regulatory cells (Tregs) and have been associated with reduced survival in humans (6–8).

Contrarily, recent evidence supports the beneficial role of B cells in cancer immunotherapy. Studies have found that the presence of infiltrating B cells are associated with favorable outcomes across multiple human cancers (9, 10). Intratumoral B cells are frequently found within organized structures analogous to those found in lymphoid organs, which are called tertiary lymphoid structures (TLS). Greater number of TLS, or expression of TLS-related genes, has been additionally associated with increased survival in patients (11–13). Cabrita and colleagues showed that B cells within TLS have high expression of MHC I and MHC II, suggesting that these B cells are capable of presenting antigens (11). Several groups have also shown that the presence of TLS is associated with response to immune checkpoint blockade (11, 13, 14). Bruno et al., showed that infiltrating B cells from human Non-small cell lung cancer (NSCLC) tumors were able to present to and activate CD4+ T cells in the presence of human tumor antigens. In addition, some patients had tumor infiltrating B cells (TIL-Bs) that could activate CD4+ tumor infiltrating lymphocytes (TILs) without exogenous antigen. When B cells from patients with NSCLC were co-cultured with CD4+ T cells, the CD4+ T cells took on a Th1 phenotype, whereas B cells with exhaustion markers yielded CD4+ T cells with a regulatory T cell phenotype (15). Taken together, these data suggest that activated B cells within TLS may act as APCs and elicit a recall response among T cells that were primed in lymph nodes, promoting their survival and proliferation, and allowing them to respond better to checkpoint blockade. This makes the function of B cells as APCs a potentially important and understudied process for cancer immunotherapy.

## 2 Antigen presentation

Generally, antigen presentation is a critical step for the immune system to generate a specific immunogenic response against a pathogen or cancer-associated tumor antigen. Typically, pathogenic/tumor specific proteins are digested by the immunoproteasomes and the resulting antigenic peptides are then loaded onto MHC molecules. They are transported to the APC cell surface for interaction with T cell receptors on T cells (16–18). This process varies widely depending upon the pathogen/tumor type and the cell type that is processing the antigenic protein. All nucleated cells are APCs, but only a few subsets are professional APCs, as they specialize in priming and expanding antigen-specific T cells (19). Another major difference between professional and non-professional APC is that MHC type II complexes are exclusively expressed on the surface of professional APCs along with MHC type I, whereas non-professional APCs only express MHC type I. Professional APCs include dendritic cells (DCs), monocytes, macrophages, and B cells, whereas all other cell types that express MHC I molecules are considered non-professional APCs (20–22). Generally, T cells are tolerant to interactions with self-antigen, as they undergo negative selection during their development in which most self-reactive high-affinity T cells undergo apoptosis. However, in the case of interaction with a foreign antigen, T cells get activated and can undergo clonal expansion to generate an adaptive immune response (23–25).

Antigens can either be directly presented or cross-presented depending upon the cell types that are involved in the process. Direct presentation is when a professional APC encounters and processes a pathogen to generate antigens and interact with T lymphocytes (26). On the other hand, if the antigen being presented on the MHC I has been internalized, but not generated by the professional APC itself, then this is called cross-presentation (27). There are several ways by which an antigen can be cross-presented, one of the most common mechanisms being internalization of free-floating antigen from the extracellular matrix, immunoproteasome processing, and then loading onto MHC I molecules for presentation (28). Other mechanisms include acquisition of pre-processed antigen from non-professional APC, which can happen either by cell-cell interaction or by cytosolic secretion of peptides. Another mechanism is by internalization of peptide-loaded MHC molecules from infected or apoptotic cells in the vicinity of a professional APC, a process also known as cross-dressing (29). Most of the cross presentation in humans is accredited to DCs (28), which survey their surroundings and capture any foreign protein/antigen from the peripheral tissue to cross present. Cross presentation is crucial in certain cases, such as viral infections that do not infect professional APCs, or tumors of non-hematopoietic origin. DCs are best suited for this task as they have been shown to have less lysosomal protease activity compared to other professional APCs (30). This results in slower degradation of the antigen and provide a safe storage space (endosomal compartment) that provides increased stability and half-life for the antigens (31).

Like DCs, B cells also participate in antigen presentation, and this can occur through direct or cross presentation. Antigenic peptide-loaded B cells interact with CD4+ as well as CD8+ T cells, leading to their activation and resulting in Th1 and Th2 type immune responses (32–34). B cells have also been shown to cross present while residing in the secondary lymphoid organs. They can encounter small soluble antigen in the lymphatic fluid that passes through the subcapsular sinus to the follicles (35). Follicular B cells can also interact with large antigens and immune complexes in the macrophage-rich subcapsular sinus, that are presented on the surface of follicular DCs (35). Some B cells that migrate through the lymph nodes can encounter antigen presented on the surface of resident DCs or newly migrated DCs located around high endothelial venules in the paracortex (36). Although it is believed that B cells are relatively weaker when compared to DCs in context of antigen presentation, some studies have shown that when B cells are exposed to certain stimuli their antigen presentation capacity can be significantly enhanced to match that of dendritic cells (32, 34, 37–39). These agents and their effects on B cells are discussed in more detail in Section 6 below.

## 3 B cell populations

B cell populations can be generally divided in to three groups, B1 cells, B2 cells and B regulatory cells (Bregs). B cells are generated throughout the human lifespan (40), and a majority of those B cells in adult humans are conventional B cells, also referred to as B2 B cells. B1 cells and Bregs constitute minor populations. In this section, we discuss these B cell types, and what is known about the APC function of these different populations.

### 3.1 Conventional B cells (B2)

B2 cell development and maturation is fundamentally categorized by the generation of an antigen-specific and mature BCR, along with other cell surface markers that define the B cell differentiation stage (**Table 1**). The BCR is the primary source of antigen presentation by B cells. Once a functional BCR is present, the cell starts expressing IgD molecules along with IgM on its surface and becomes a naïve mature B cell, which then leaves the bone marrow and migrates to secondary lymphoid organs such as spleen and lymph nodes (41). Immature B cells transit to spleen where they attain maturation and activation. This process can also be divided into several stages depending upon the location and activation status of the cell. B cells migrating from the bone marrow to the spleen are known as transitional B cells (42). In the spleen, B cells can be classified as marginal zone B cells, regulatory B cells, follicular B cells, activated B cells, germinal center B cells, plasma cells (short or long lived) and memory B cells (43). These B cell subsets can also be identified based on the differences in the expression of certain cell surface and intracellular protein markers, as listed in **Table 1**.

**Table 1:** Expression profile of cell surface and intracellular markers characteristic of developmental stages of B cells in humans and mice.

Developmental Stage	Mouse	Humans
**Common Lymphoid Progenitor**	Lin- CD117- Ly6- Ly6D- IL-7 Ra+ Flt-3+	CD10+ CD34+ Pax5+
**B lymphocyte Progenitor**	Lin- CD117- Ly6- Ly6D+ IL-7 Ra+ Flt-3+	CD10+ CD34+ Pax5+
**B1 Progenitor**	Lin- CD45R low CD19+ CD93+	–
**B2 Progenitor**	Lin- CD45R+ CD19- CD93-	–
**Pre-Pro B cell**	Lin- CD45R+ CD19- CD24 low CD43+ CD93+ CD117- CXCR4+ FLt-3+ IL-7Ra+ IgM-	CD117 low CD10+ CD34+ CD38+ Pax5+
**Pro-B cells**	Lin- CD45R+ CD19+ CD24 + CD43+ CD117 low IL-7Ra+ IgM-	CD117 low CD10+ CD19+ CD20+ CD24+ CD34+ CD38+ CD93+ IL-3R+ IL-7 Ra+ Pax5+
**Pre-B cells**	Lin- CD45R+ CD19+ CD24 + CD43- IL-7Ra+ IgM-	CD117- CD10+ CD19+ CD20+ CD24+ CD34- CD38+ CD93+ IL-3 R+ IL-4 Ra+ IL-7 Ra+ Pax5+
**Immature B cells**	CD45R+ CD19+ CD23- CD24+ CD43- CD93+ IgD- IgM+	CD117- CD10+ CD19+ CD20+ CD21+ CD24+ CD27- CD38+ CD40+ CD93+ IL-4 Ra+ IL-7 Ra-
**B1a Cells**	CD1d mid CD5+ CD19 high CD23- CD43+	–
**B1b cells**	CD1d mid CD5- CD19 high CD23- CD43+	–
**Transitional B cells**	CD45R+ CD19+ CD24+ CD43- CD93+ IgM+ IgD low/+ (T1/T2)	CD10 low CD5+ CD19+ CD20+ CD21+ CD23+ CD24+ CD27- CD38+ CD93+ TACI+
**Marginal Zone B cells**	CD45R+ CD1d+ CD19 mid CD21 high CD23- CD43- CD93- IgM high IgD low	CD1c+ CD19+ CD20+ CD21+ CD27+ FCRL3+ TACI+
**Follicular B cells**	CD45R+ CD1d mid CD19 mid CD21 low CD23+ CD43- CXCR5+ IgM low IgD high	CD10- CD19+ CD20+ CD21+ CD22+ CD23+ CD24 low CD27- CD38 low CXCR5+ TAC+ MHC II+
**Activated Germinal Center B cells**	CD45R+ CD19+ CD40+ MHC II+	CD19+ CD20+ CD27+ CD38+ CD40+ CD83+ TACI+ MHC II+
**Memory B cells**	CD45R low CD19+ CD21+ CD27 mid/+ CD40+ MHC II+	CD19+ CD20+ CD21+ CD27+ CD93- TACI+
**Plasmablast**	CD45R low CD19+ CD27 high CD38+ CD138+	BCMA+ CD19 low CD27 high CD38+ CD93+ CD138-/low
**Plasma cells**	CD45R low BLIMP1+ CD19- CD27 high CD38 low CXCR4 high CD138+ MHC II -/low	BCMA+ BLIMP1+ CD19 low CD20-/low CD27 high CD38 high CD138+ CXCR4+ MHC II low
**Regulatory B cells**	CD1d+ CD5+ CD19+ CD23-/low CD24+ CD93-/low TIM-1+ IL-10+ IL-35+ TGF-β+	CD1d+ CD5+ CD19+ CD21+ CD24+ IL-10+ IL-35+ TGF-β+

For CLP, BLP, B1 and B2 progenitor cells: Lin- is CD3- CD4- CD8- Gr-1- CD11b- TER-119- For PrePro B, Pro B and Pre B cells: Lin- = CD3- Gr-1- CD11b- TER-119-.

Antigen presentation by B2 cells primarily occurs through the BCR, in which interaction with a BCR-specific antigen leads to B cell activation and proliferation, and also internalization of that antigen which leads to its processing and presentation (44). However, as demonstrated by Silver et al., B2 cells have also been shown to internalize and process antigens that are not specific to the BCR when exposed to them at high concentrations (45). Although, if their findings are relevant in real physiological world or not remains unknown, the property of B cells to uptake non-cognate antigen *via* a BCR-independent pathway can be of relevance. Moreover, B cells can also be activated through germline-encoded PAMP (pathogen associated molecular pattern) receptors, by a phenomenon known as polyclonal B cell activation (46, 47). The impact of BCR and PAMP receptors on the APC function of B cells is discussed in more detail in Section 4.

Classically, upon BCR encounter with cognate antigen in the spleen or lymph nodes, B2 cells undergo activation, class switching, division and proliferation. Class switching, or isotype switching, occurs when naïve B cells switch from expressing IgM and IgD to other isotypes such as IgA, IgG, IgE (48). This is determined by the type of cytokine signal B cells receive from T helper cells after presenting antigen. IL-4 (49), IL-5 (50), TGFβ (51) and IFNγ (52) are all known to induce isotype switching after activation in mice and humans. The two most prominent cell subtypes that occur after differentiation of B cells are plasma cells, that produce antibodies against the antigen/pathogen, and memory B cells, that can respond quickly to subsequent exposure to the same antigen (53). Plasma cells are generated in great numbers and are short lived, whereas the memory cells are long lived and are generated in small numbers.

B2 cells are protected from foreign antigens as they undergo development in the sterile environment of the bone marrow. Consequently, their role as APCs becomes of importance once they become transitional B cells and transit to the spleen and other lymphoid organs. They are effectively shielded from interaction with foreign antigen(s) as they are not matured. B2 cells express most of the machinery that is required for antigen processing and presentation, such as MHC II, invariant chain (li), calnexin and HLA-DM, during their early developmental stages (Pro-B, Pre-B and immature B cells). However, a mature BCR is absent, along with HLA-DO and CLIP (54–56). Once the immature B cells leave the bone marrow and are in the transitional stage, that is the first opportunity for them to naturally encounter and respond to a foreign antigen (57). Some reports suggest that when transitional B cells encounter an antigen they are marked for apoptosis, in contrast to mature B cells which get activated upon antigen encounter (58, 59). But a recent *in vitro* study suggested that both T1 and T2 transitional B cells can process and present antigen just as well as mature B cells, but require survival signals from pre-activated CD4 T cells (57, 60).

Expression of HLA-DO and proteolytic cleavage of invariant chain (li) to CLIP peptide are initiated only in naïve follicular B cells and their subsequently differentiated B cell subtypes. Expression of MHC II related antigen presentation components are lost in terminally differentiated antigen-specific plasma cells, and these cells cannot present antigen or undergo class switching (61, 62). Immature plasmablasts are capable of antigen presentation until they mature and become fully differentiated plasma cells (63). Mature naïve B cells are known to encounter antigen in the lymphoid follicles. T cells in the germinal centers help B cells in the process of activation, which is followed by class switching, somatic hypermutation and finally clonal differentiation (64). There is not much known about which subtype(s) of B cells can present antigen through the MHC I pathway, as traditionally B cells are known to present through the MHC II pathway to CD4 T cells, and most research has focused on that. But based on the observation that MHC I is ubiquitously expressed on B cells that express MHC II (65), it is likely that all B cell subtypes discussed above that can present through MHC class II pathway should also be capable of presenting antigen to CD8 T cells *via* MHC I pathway.

### 3.2 B1 cells

B-1 cells can be characterized as either B1a or B1b B cells on the basis of expression of Ly-1 (CD5) on their surface (66). B1a B cells express CD5 and B1b B cells do not, while expression of other cell surface markers is common to both, as in **Table 1**. Both of these types predominantly develop in the fetal liver, and their functions are classified as innate immune type responses (67). B1 B cells can also develop during adulthood in the bone marrow, but their frequency is very low in comparison to B2 B cells (68). B1 cells primarily secrete IgM, but also IgA and IgG3, without any antigenic stimulus. Hence these antibodies are called natural antibodies, as they have high reactivity but low affinity towards pathogens (69). The natural antibodies are secreted independent of T cell help and are the first barrier against a pathogenic infection, before the development of an adaptive response (70, 71). About 80% of all naturally occurring IgM is secreted by B1 cells, which is thought to maintain immune homeostasis, by regulating B-1 cell development as a feedback mechanism along with regulation of IgG2a production and promotion of B2 cell antibody responses (72, 73). Some studies have also correlated increased numbers of B1 cell populations with development of autoimmune diseases, such as in patients with Sjogren’s syndrome (74) and rheumatoid arthritis (75).

The function of B1 B cells as APC has also been studied in less detail compared to B2 B cells. As previously discussed, B1 B cells are known for their ability to generate an innate type immune response following activation by T-independent antigens (67, 69). Some groups have also demonstrated their ability to generate T-dependent immune responses (76–81). B1 B cells mostly function independent of T cell signals/antigens, however, they can present antigen to T cells *in vitro* (78) and *in vivo* (76, 80, 81). After adoptive transfer of OVA-peptide pulsed B1a B cells along with CFSE labeled OVA-antigen specific CD4 T cells in mice, Margry et al., showed that B1a B cells were able to induce proliferation of antigen-specific CD4 T cells (76). This fact is also supported by the fact that B1 B cell subsets can constitutively express markers associated with antigen presentation and co-stimulation, such as MHC II, CD80 and CD86, upon stimulus (78). Moreover, reports from Zimecki et al., indicate that B1 B cells may be superior to conventional B2 cells in terms of antigen presentation, as they elicit greater proliferation of antigen-specific T cells (80, 81).

The activation of B1 B cells following BCR-antigen interaction or TLR ligation is tightly regulated. This is because B1 cells express BCR that can cross-react with self-antigens (82). Several mechanisms are involved in suppressing the activation of B1 cells after BCR engagement. Recent studies have shown that BCR and TLRs play critical roles in regulating antigen uptake by B1 cells (82–84). However, some reports demonstrate macrophage-like phagocytic function of B1 cells, that enables these cells to function as APCs (85). Moreover, it was demonstrated that the phagocytic function was enhanced in the presence of an antigen specific BCR. However, this does not eliminate the possibility of BCR-independent phagocytic pathways in B1 B cells that may include scavenger receptors, complement receptors, Fc receptors and integrins (85). Their phagocytic potential can be further augmented by use of adjuvants such as *Propionibacterium acnes* (86) and LPS (87, 88). Whether these stimuli also promotes their antigen presentation activity, is not clear and is currently under investigation.

### 3.3 Regulatory B cells (Breg)

Bregs are a minor B cell population, typically comprising of less than 1% of human peripheral blood mononuclear cells (PBMC), and are characterized by their immunomodulatory function to suppress inflammation (89). Most B cell subsets are capable of differentiating into Breg cells to regulate inflammation, and usually do so through IL-10 secretion (90), or IL-35 secretion (91). CD138+ plasma cells have also been shown to produce IL-10 and IL-35, and can differentiate into Bregs (91). TGF-β has also been shown to be produced by Breg cells to mediate induction of Tregs during tolerance induction after transplantation (92).

Within the last decade, several subsets of Bregs have been identified in mice and humans based upon differential expression of cell surface markers. A list of their expression profiles and associated phenotypes are listed in **Table 2**. Several B cell subsets, such as immature B cells, plasmablasts and mature B cells, have been shown to differentiate into IL-10-producing Bregs, upon stimulation with toll-like receptors (TLRs) and/or CD40 activation (93, 94). Differentiation of B cells into Bregs can also occur after stimulation of B cells through BCR signaling, following antigen encounter and presentation (95), and when this happens the resulting Bregs are antigen-specific (96). As mentioned previously, IL-10 is one of the major factors used to discriminate a Breg reliably from a conventional B cell, but their identification by cell surface markers has been controversial. As there is no known transcription factor or lineage-specific marker for Bregs (unlike FoxP3 as a marker for Tregs), it has been difficult to identify and define Breg populations. Although Bregs are known to indirectly suppress antigen presentation by DCs monocytes and macrophages, to our knowledge they have not been tested for their ability to prime and activate T cells through antigen presentation (97–99). Given their intrinsic characteristics they would likely be poor candidates for priming and activating T cells.

**Table 2:** Expression of cell surface markers in different phenotypes of Breg cells and their function in mice and humans.

*2a. Mice*			
Breg Type	Expression Profile	Location	Function
*2b. Humans*			
Breg Type	Expression Profile	Location	Function
**T2-MZP cells**	CD19+ CD21 high CD23 high CD24 high	Spleen	IL-10 production, induction of Tregs, suppression of CD4 and CD8 T cells
**MZ cells**	CD19+ CD21 high CD23-	Spleen	IL-10 production, induction of Tregs, suppression of CD4 and CD8 T cells
**B10 cells**	CD5+ CD1d high	Spleen	IL-10 production, induction of Tregs, suppression of CD4 T cells, monocytes and DCs
**Plasma cells**	CD138+ MHCII low B220+	Spleen	IL-10 and IL-35 production, suppression of NK cells and effector CD4 T cells
**Tim-1+ B cells**	Tim-1+ CD19+	Spleen	IL-10 production and suppression of effector CD4 T cells
**Plasmablasts**	CD138+ CD44 high	Draining Lymph Nodes	IL-10 production and suppression of effector CD4 T cells and DCs
**B10 cells**	CD24 high CD27+	Blood	IL-10 production, suppression of DCs, monocytes, and effector CD4 T cells
**Plasmablasts**	CD119+ CD24 high CD27 int	Blood	IL-10 production, suppression of DC and effector CD4 T cells
**Immature cells**	CD19+ CD24 high CD38 high	Blood and site of inflammation	Production of IL-10, induction of Treg, suppression of Th1 and Th17, support iNKT homeostasis
**Br1 Cells**	CD19+ CD25 high CD71 high	Blood	Production of IL-10 and IgG4

## 4 Antigen processing and presentation by B cells

Activation of B cells as APCs occurs *via* two major pathways: (i) through the B-cell receptor (T-cell dependent), or (ii) through germline encoded PAMP receptors (T-cell independent). PAMP receptors for macromolecules like polysaccharides, lipopolysaccharides, and other non-protein antigens, have been shown to induce antigen presentation in B cells. The BCR specifically interacts with its cognate antigen, inducing a signaling cascade that stimulates internalization of the antigen, leading to B-cell activation and proliferation. Once interacting with membrane-associated antigen, such as antigen presented by follicular dendritic cells or antigen on pathogenic cells, B cells exhibits a membrane spreading and contraction response that assists with BCR signaling and antigen gathering. This results in the formation of an immunological synapse at the B cell plasma membrane region contacting membrane-associated antigen (18, 56, 100–103). There are several mechanisms that are involved in BCR-mediated antigen internalization including clathrin-mediated endocytosis, fast endophilin-mediated endocytosis, and caveolin-dependent endocytosis (104). However, unique to naïve B cells, BCR-mediated antigen internalization and processing is mediated by Cbl and Cbl-b (Cbls), which belong to the superfamily of E3 ubiquitin ligases. Cbls promote naive B cell conversion into mature antigen presenting B cells and are essential for interaction between naïve B cells and cognate T cells (105).

As discussed earlier, the BCR plays an important role during the development of B cells, but is also crucial when presenting antigens. When the BCR encounters its cognate antigen, B cells are signaled to proliferate and differentiate into traditional antibody-secreting plasma cells and long-lived memory cells. Upon antigen binding, BCR oligomers are formed and the affinity of the antigen towards the BCR regulates the strength of BCR signaling (106). Tsourkas et al., demonstrated that as the affinity increases, the size and rate of oligomer formation as well as the number of antigens collected by the BCR, increases (107). Furthermore, Liu et al. demonstrated that BCR-intrinsic mechanisms enable B cells to read the affinity of the antigen in very early phases of BCR-antigen interaction and that the growth of BCR micro-clusters was dependent on the antigen affinity (108). BCRs also mediate endocytosis of the antigen by receptor internalization, for further processing and presentation to helper T cells. Signals through the BCR are a driving factor for B-cell survival, activation, maturation, migration and differentiation into various developmental stages (109). Variations in BCR signaling have also been shown to affect the expression levels of co-stimulatory molecules on antigen-activated B cells.

### 4.1 Antigen uptake and processing

Professional APCs typically use one of the three major pathways for antigen internalization: endocytosis, pinocytosis, or phagocytosis (20). Generally, naïve B cells do not have phagocytic capabilities, but B1-B cells have been shown to phagocytose particles, including bacteria (85). Another recent report demonstrated that cognate follicular B cells can mediate phagocytosis of particulate antigens through the BCR, which is mediated by RhoG GTPase (110). However, the majority of antigen acquisition by B cells occurs through endocytosis for antigens smaller than 0.2µm (22). We have also demonstrated that B cells can acquire antigen through fluid phase pinocytosis when plasmid DNA is used as an antigen (111). Antigenic peptides generated after either of these internalization pathways can be presented but can have different efficiencies, depending upon various factors such as antigen type, internalization mechanism used for antigen uptake, and APC subset (B cells, DCs, macrophages) involved in antigen presentation.

Processed antigens are presented on major histocompatibility complexes (MHC) which are assembled in the endoplasmic reticulum and functionally matured in the endosomal compartments (112). As previously discussed, there are two types of MHC molecules; class I and class II; MHC I complexes are generally loaded with antigenic peptides that results from proteasomal degradation of cytosolic proteins. In this pathway, cytosolic protein could be a self-protein, a viral protein (from a virus that has infected the cell) or an exogenous protein resulting from retro-translocation after phagocytosis of a pathogen. After proteasomal degradation, the peptides are transported to the ER through TAP, then processed by ERAP to be loaded on MHC molecules, and finally transported to the cell surface for presentation (113). Most exogenous proteins get processed and loaded on MHC II complexes in late endosomal compartments (114). Some cytosolic proteins are also processed through this pathway during autophagy (115).

Complement-coated antigens presented to B cells remain on the cell surface, bound by complement receptors, whereas IgG-coated antigen received by FcγRIIB receptor gets internalized in neutral endosomes and recycled to the surface either for presentation to DCs (cross presentation) or to T cells by MHC II (116). Marginal zone B cells act as antigen transporters and can carry complement-coated antigens to follicular DCs in the spleen or lymph nodes (117). This transportation is not mediated by BCRs, so the B cells need not be antigen specific. Complement receptors, CR1 and CR2, act as transporters in this situation (117).

When BCR is involved in antigen detection and capture, antigen processing takes place through the traditional MHC II loading pathway. B cells express clonally specific BCR which allows them to capture antigen even at low concentrations. This is unlike other professional APCs which require comparatively larger amounts of antigen for consequent activation and presentation. Moreover, higher affinity antigens generate stronger and rapid BCR signaling and have greater efficiency in presenting to T cells (118). There are some unique features that have only been reported when B cells present antigen through the MHC II pathway. According to some reports, MHC II localizes to unique MHC class II vesicle compartments that are distinct from lysosomes and endosomes (119). Upon ligation of BCR by cognate antigen, MHCII dimers are redistributed to LAMP1-positive multivesicular bodies (103). BCR-antigen complexes are then transported through the endocytic pathways to MHC II-rich regions where ubiquitinylation occurs. E3 ligase “Itch” mediates ubiquitinylation of Igβ (120), which is important for sorting the complexes to LAMP1-positive compartments. In these compartments HLA-DM mediates loading of antigenic peptide by removal of CLIP peptide from MHCII (121), and also distinguishes between strong versus weak binders from the peptide repertoire. Preferential loading of peptides that fit more tightly ultimately generates a highly specific response (122). As reported by Lankar et al., the dynamic changes following BCR stimulation in non-activated mature B cells are transient and reversible after 24 hours (123).

Expression of HLA-DO in B cells also regulates antigen presentation (124) by inhibiting expression of HLA-DM (125). Generally, germinal center B cells that are competent APCs have diminished expression of HLA-DO (126). B cells downregulate the expression of HLA-DO only when MHC II localizes in acidic compartments, which allows for HLA-DM-dependent peptide loading. This also maintains specificity for peptide loading of processed antigen that are internalized through the BCR (127).

Apart from presentation through MHC II complex, B cells can also process and present antigen through MHC I complexes, but less is known about this. There are several reports that show antigenic-peptide pulsed B cells are capable of presenting antigen to CD8 T cells, but are not as efficient as dendritic cells when it comes to activation and proliferation of antigen-specific CD8 T cells (128, 129). The specific details of the mechanism by which B cells process and load antigens onto MHC I complexes, rather than onto MHC II complexes, are not clearly understood.

### 4.2 Activation and co-stimulatory signals

Generally, three signals are required to activate T cells (130), the first of which is in the form of the antigen being presented by the APC. Interaction of antigen-loaded MHC with the T-cell receptor initiates signaling that activates T cells for their proliferation and differentiation (131). Secondly, the T cell needs to receive a costimulatory signal, which occurs primarily by CD80/CD86 on the APC binding to CD28 on the T cell. This second signal stabilizes the immunological synapse between APC and the T cells, and induces the expression of other activation markers. Upon activation, B cells express MHC class I and II, CD80, CD83, CD86, CD40 and other costimulatory and adhesion molecules that support and strengthen antigen presentation (132). Finally, the T cells receive the third signal in the form of cytokines that activate them and polarize them towards an effector phenotype. In the case of B cells as the APC, B cells secrete CCL22 and CCL17 upon activation to recruit CD4 T cells, which in turn secrete cytokines such as IL4 to promote B cells differentiation towards cytokine secreting effector cells (133, 134). Upon differentiation cytokines secreted by B cells can include IL-2, IL-4, IL-6, TNFα, and/or IFNγ which can have polarization/inhibition effects on the immune response (135).

Many of the costimulatory and/or activation signals promote differentiation, survival and proliferation of both B cells and T cells. But some of these are unidirectional and act on either B cells or T cells alone upon encounter with their ligand. Most important of all costimulatory/activation interactions is CD80/CD86 (APCs) and CD28 (T cells), as this interaction is involved with establishment of the immune synapse (136). Other cell surface interactions take place between APCs and T cells and occur after the presentation of antigen to T cells. For example 4-1BB, ICOS and OX40 all become expressed on T cells after T-cell activation, whereas their ligands are present on activated APCs along with CD80/CD86 even before T-cell activation (137).

The roles of CD80 and CD86 have been extensively studied in the context of antigen presentation and it has been shown that increased expression of either of these make B cells potent APCs, as they provide stronger intercellular interaction and a more stable environment for T cell activation (136). CD80 and CD86 also play an important role in antibody secretion; one study demonstrated that an antibody targeting CD80 in LPS activated B cells can suppress IgG secretion whereas an antibody targeting CD86 promotes antibody secretion (138). Signaling through CD83, on the other hand, is less understood. The identification of CD83L on T cells has been controversial. A few studies propose that a receptor for CD83 is expressed on human and murine CD4 and CD8 T cells (139). Expression of CD83 on B cells marks their activation, especially during germinal center reaction (140). It was also demonstrated by Akauliya et al., that antibody responses to influenza infection were significantly lower in CD83 KO mice compared to wild type mice, implying a role of CD83 in modulation of antibody responses, but this could also be attributed to reduced numbers of CD4 T cells in CD83KO mice (141). In another study using CD83 knock out mice, Krzyzak et al., reported defects in MHC II and CD86 expression upon stimulation, results in modulations in germinal center composition (more B cells in dark zones) and an enhanced IgE response (140).

Other costimulatory molecules expressed on B cells include ICOSL, CD134L (OX40L) and CD137L (4-1BBL) (**Table 3**). Each ligand interacts with its specific receptor and contributes towards B-cell and/or T-cell co-stimulation. ICOS (inducible co-stimulator) binds with ICOSL (ICOS ligand) and provides both positive and negative co-stimulatory signals to B cells. Their interaction promotes B-cell activation and differentiation. More specifically, it has been demonstrated that ICOS signaling promotes antibody-secreting B cells (142). It has also been shown that in ICOS-deficient mice, its absence impairs germinal center formation and causes defects in contact-dependent isotype class switching (143). Similarly, interaction between OX40 and OX40L promotes B-cell activation, proliferation, survival, and cytokine production (144, 145), but if disrupted/inhibited it can cause reduction in production of class-switched immunoglobulin isotypes (146). In contrast, CD137-CD137L interaction stimulates T cells only, as CD137 is only expressed on activated T cells (147, 148). There has not been any evidence that suggests a critical role of 4-1BB-4-1BBL signaling in B-cell activation or development.

**Table 3:** Expression of co-stimulatory and activation molecules on B cells during antigen presentation.

Receptor	Ligand	Function
CD80/86	CD28 and CTLA4	T cell activation and survival (CD28), inhibitory regulation of activated T cells (CTLA4)
CD83	CD83L	Prolonged expansion of CD8 T cells
CD278L (ICOSL)	CD278 (ICOS)	Stimulation and proliferation of T cells
CD134L (OX40L)	CD134 (OX40)	Stimulation and promotion of IgG response
CD137L (4-1BBL)	CD137 (4-1BB)	Stimulation of effector T cells
CD40/CD40L	CD40L/CD40	Activation, maturation, differentiation, proliferation, isotype switching, survival, cytokine production, memory response development
CD22	N-linked oligosaccharides	Adhesion and BCR signaling modulation
CD81	No natural ligands known yet	Credited towards adhesion and T cell-dependent B cell activation
CD11a-CD18/CD54(LFA1/ICAM1)	CD54/CD11a-CD18(ICAM1/LFA1)	Cell adhesion and enhanced activation & antigen presentation
CD72	CD100	Enhanced antigen presentation, development of B1b cells and production of high affinity IgG response

One of the most studied costimulatory interactions between B cells and T cells is CD40-CD40L. The receptor and ligand can each be expressed by either cell type, however, CD40 is primarily expressed by B cells and CD40L is primarily expressed on activated T cells. Activated human and mouse B cells have been shown to express CD40L on their surface, which can be secreted in its soluble form for use as an autocrine ligand (149). In some special cases, such as in a unique pathogenic CD4 T cell population in type1 diabetes and during CD8 T cell memory generation, T cells can express CD40 (150, 151). Most of the effect resulting from this interaction occurs in B cells, as it can promote activation, cytokine production, proliferation, antibody secretion and upregulation of several surface molecules involved in antigen presentation (152, 153). Evidence shows that CD40-CD40L signaling also regulates class switching, formation of germinal centers and humoral memory response (154). Most studies that have evaluated the role of CD40 signaling in B cells have shown that activation of CD40 plays a critical role in presenting antigen and activation of antigen-specific T cells. CD40 activation also results in improved survival of B cells through CD40-induced phosphoinositide 3-kinases (155).

Adhesion molecules such as ICAM-1 and LFA-1 (**Table 3**) are also expressed on B cells during antigen presentation; their interaction ensures increased stability of the synapse and results in amplification of activation signals (156). Specifically in the case of B cells, this signaling has been shown to promote antigen presentation by B cells by cooperating with CD40 signaling (157). Another adhesion molecule, CD22, belonging to the immunoglobulin superfamily, is also expressed on B cells. Several natural ligands, specifically N-linked oligosaccharides, are known to interact with CD22, many of which can be expressed on T cells and can potentially interact with CD22 (158). The exact nature of this interaction is not clearly understood as specific receptor-ligand pairs between B cells and T cells that involve CD22 are not known. Another surface molecule, CD81, which belongs to the tetraspanin family, has also been reported to contribute towards B-cell adhesion and T-cell dependent activation (159), but its natural ligand(s) are yet to be discovered.

## 5 Role of CD4 T cells in antigen presentation by B cells

In addition to effects mediated by co-stimulatory and adhesion molecules, B-cell activation and function can also be modulated by CD4 T cells and other environmental factors such as cytokines and TLR ligands. The interaction between B cells and CD4 T cells is very well studied, but mostly in the context of antigen presentation through the BCR and adaptive immunity – their role in presentation of antigen *via* MHC I is less studied (32, 160, 161). This interaction results in affinity maturation and differentiation of B cells into plasma cells and memory cells, leading to antibody secretion and at the same time regulating the development of memory CD4 T cells (161, 162). One of the most important interactions between CD4 T cells and B cells is the CD40-CD40L (B cell – CD4 T cell) interaction, which plays a crucial rule in activation of B cells. As discussed earlier, activation of the CD40 pathway in B cells leads to a multitude of responses that are required by B cells for survival, activation and proliferation (162, 163). Upon stimulation by B cells, CD4 T cells start secreting IL-2, which primarily acts as an autocrine differentiation and proliferation factor, and also promotes the development and maintenance of Tregs (164, 165). However, IL-2 has also been shown to affect B cell proliferation, specifically in humans (166), and induce differentiation of activated B cells into plasma cells (167).

A subset of CD4 T cells, called the T follicular helper cells, promote B cell proliferation and effector function through the production of IL-4 and IL-21 (168, 169). Studies have shown that knockout of either of these two cytokines leads to diminished B cell responses, which are further diminished with a combined deficiency of both (170). More specifically, the IL-4 pathway is primarily involved in the formation of germinal centers in type 2 immune responses (64, 171), whereas IL-21 influences the differentiation of B cells into Ig-secreting plasma cells (172, 173).

## 6 External agents that can affect antigen presentation

### 6.1 Cytokines

Like all immune cells, B cells are influenced by external stimuli provided by cytokines. Pro-inflammatory cytokines support antigen presentation through B cells by upregulating the co-stimulatory molecules. IL-4 induces a 10-fold increased expression of class II MHC antigen on B cells, and stimulation with IL-21 alone, or IL-21 and IL-2, upregulates CD86 expression on B cells (174, 175). Other studies have demonstrated the importance of IL-4 and/or IL-21 in promoting antigen presentation, specifically in B cells. IFN-γ is also thought to facilitate the antigen-presenting activity of B cells; although some studies report that IFNγ downregulates the cell surface expression of MHC II on B cell in PBMC (176) and cell lines (39), while upregulating MHC I expression. Treatment with IFN-γ can also regulate the proliferation and differentiation of B cells (177, 178). IFN-γ has both positive and negative effects on B cell proliferation depending upon the stage of antigen presentation. Before antigen encounter, and in the later stages of antigen presentation, IFN-γ inhibits proliferation. However, during the early proliferative response upon antigen encounter, IFN-γ promotes B cell division. IFN-γ has also been reported to mediate and regulate antibody class switching on B cells. Interestingly, some anti-inflammatory cytokines can also support antigen presentation activity of B cells. IL-13 has been reported to enhance the expression of MHC II on B cells, and TGF-β also modestly increased the expression of MHC class II on B cells (179, 180).

### 6.2 Toll-like receptors (TLRs)

In addition to cytokines, the antigen presentation activity of B cells is also influenced by TLRs. TLRs are type I transmembrane receptors which sense molecules containing PAMPs or Damage Associated Molecular Patterns (DAMPs). These receptors are expressed in B cells and affect their antigen presentation activity (181, 182). It was shown that TLR9 stimulation facilitates B cell antigen presentation with the upregulation of MHC class II, CD40, and CD80. Similarly, TLR2 and TLR4 stimulation also increase the expression of CD86 and MHC II in B cells (183–185). TLR7/8 ligands have also been reported to upregulate CD80 expression on B cells (186). TLR9 has been of most interest in the context of stimulating B cells for antigen presentation, and many studies show that TLR9 stimulation by CpG ODN (oligodeoxynucleotides) leads to increased activation of B cells that promotes both innate and adaptive immune responses (38, 187–190). Jiang et al., reported that stimulation of naïve B cells with CpG ODN rescued them from apoptosis, caused proliferation and enhanced the expression of CD40, CD80, HLA-DR on their surface (191). They further demonstrated that these B cells do not mature into memory cells, but rather have increased ability to activate allogeneic CD4 and CD8 T cells (191). This can also be further enhanced when B cells are treated with a combination of TLR9 agonist and other stimulating agents (192–194). As reported by Giordani et al., IFN-alpha amplifies the effects of TLR9-mediated activation of naïve B cells. They demonstrated that there was increase in B cell activation, Ig production and frequency of CpG-induced memory B cells (193).

### 6.3 Other proteins

BAFF (B cell-activating factor) is fundamental for B cell survival and maturation (195). It has recently been found to upregulate B cell expression of CD40 and enhance B cell antigen-presentation to CD4+ T cells through increased expression of MHCII (196). In a mouse melanoma model, treatment with recombinant BAFF promoted central memory phenotype of T cells in vaccine-draining lymph nodes, along with an increase in the number of B cells with upregulated costimulatory molecules (197). Treatment with BAFF led to downstream T-cell activation and increased anti-tumor immunity, demonstrating one method of converting B cells into highly effective APCs. Along with T cell mediated anti-tumor effects, BAFF also induced CD4+FoxP3+ Treg population in the spleen and tumor microenvironment (197). However, overexpression of BAFF has been associated with autoimmune diseases in mice and humans, by allowing the emergence of autoreactive B cells (198, 199).

## 7 Outcome of antigen presentation

The outcome of antigen presentation on T cells, in terms of their phenotype and effector function, can vary vastly based on the antigen internalization pathway, the form of antigen presented (DNA/RNA/protein/peptide), the APC subtype involved, and the activation/developmental stage of the APC, as any of these factors can impact the efficacy of antigen processing and presentation. Consequently, here we discuss what is known about the effects on T cells activated by antigen presenting B cells, specifically related to CD8 T cells, how B cells may differ from other professional APCs, and what implications this has on use of B cells as APCs in immunotherapy.

### 7.1 Direct and cross presentation

One study, conducted in a Salmonella infection model, showed that antigen-specific B cells are capable of cross-presenting antigen to CD8+ T cells, and this cross-presentation is partially dependent on proteasomes; CD8+ T cells demonstrated decreased degranulation, as measured by CD107a expression, when CD8+ T cells were primed by proteasome-inhibited B cells (200). Salmonella-infected B cells were shown to promote CD8 T-cell proliferation with the help of CD4 T cells, and the resulting CD8 T cells were cytotoxic and secreted IFN-γ. Wit et al., also demonstrated that Salmonella-infected B cells could activate both central and effector memory CD8 T cells (200). Two other studies have demonstrated enhanced B cell cross-presentation when antigen is delivered with adjuvants. B cell cross-presentation to cytotoxic CD8+ T cells was enhanced when antigen was co-delivered with CpG-DNA (38) or a TLR2 agonist (201).

A lesser known and understudied function of B cells is the direct presentation of antigen to CD8 T cells on MHCI. Zentz et al., demonstrated that CD40-activated B cells (via co-culture with CD40L-expressing irradiated fibroblasts) could strongly and specifically expand rare populations of antigen-specific CD8 T cells from the PBMC of healthy donors. Epitope-specific (HPV-16, E7) CD8 T cells could be selectively expanded in 6 of 6 healthy donors with initial frequencies of less than 1 in 20,000 antigen-specific CD8 T cells. The authors observed up to a 10^6^-fold expansion of antigen-specific CD8 T cells, and the resulting cultures contained up to 88% antigen-specific CD8 T cells (202). CD40-activated B cells (CD40-B cells) have similarly been used by many groups as a readily available source of highly efficient APC and have been shown to be capable of priming Th1 type anti-tumor responses (34, 187, 203, 204). In a murine study assessing the use of CD40-B cells as an anti-cancer vaccine, vaccination of wild-type mice with LCMV antigen-pulsed CD40-B cells significantly reduced growth of LL-LCMV subcutaneous tumors by direct and indirect activation of CD8 T cells, but antigen-pulsed LPS-activated B cells did not (205). Furthermore, using CD40L-expressing feeder cells for activation, vaccination with tumor antigen-pulsed CD40-B cells resulted in significantly delayed growth in both B16 melanomas and E.G7 lymphomas (206).

In related studies, CD40-B cells transduced with tumor antigen-encoding RNA or DNA have been demonstrated to prime tumor-specific cytotoxic CD4 and CD8 T cells *in vivo*. Fujiwara and colleagues generated a eukaryotic expression vector which contained three leukemia-specific antigens, primary granule protein proteinase 3, human neutrophil elastase, and cathepsin-G, inserted into the pcDNA3.1 plasmid. PBMC from five HLA-A2+ leukemia patients were cultured with plasmid-transduced CD40-B cells. The transduced B cells were able to stimulate both CD4 and CD8 responses against all three antigens. Furthermore, when CD3 cells isolated from PBMC were stimulated with DNA-transfected autologous CD40-B cells, the investigators were able to culture CD4 and CD8 T-cell lines that produced IFN-γ upon stimulation with autologous leukemia cells (207). Coughlin et al., similarly demonstrated that CD40-B cells transfected with RNA could serve as a vaccine for tumor antigens. RNA transfected CD40-B cells induced IFNγ+ cytotoxic T cells which could be identified with tetramers and lysed neuroblastoma cell lines (208). Taken together, these studies indicate that CD40-activated B cells can express, process and present antigens on both MHCI and MHCII when transduced with tumor antigen encoded by DNA or RNA.

### 7.2 Comparison of B cells to other APCs

Activated B cells have many similarities with DC in terms of APC function. For example, after three to four weeks of CD40 activation, B cells were shown to express high levels of HLA class I and the costimulatory molecules CD80 and CD86 (207). However, CD40-activated B cells differ from DCs by displaying a rapid migratory pattern and undergoing highly dynamic, short-lived and sequential interactions with T cells (209). Previous work from our group indicates that short APC to T-cell contact times can stimulate T cells with transient PD-1 expression, while longer (>15min) contact times resulted in persistent PD-1 expression and attenuated anti-tumor responses (210). Taken together these data suggest that T cell/B cell interactions could prove advantageous over T cell/DC interactions when initiating a cytotoxic response against PD-L1+ target cells (e.g., in many solid tumors) by stimulating T cells that may be less susceptible to PD-1 ligation. This remains to be demonstrated, but conceptually B cells may differ from DC in inducing expression of other T cell checkpoint molecules on activated T cells. Furthermore, the tumor microenvironment is filled with other immunosuppressive tumor-derived factors, such as, prostaglandin E2 (211), TGF-β (212), VEGF (213) and IL-10 (214) which act in part by inhibiting DC differentiation, maturation, trafficking, and antigen presentation (215). Activated B cells, on the other hand, are relatively resistant to inhibition by tumor-associated immunosuppressive factors such as IL-10, TGF-β and VEGF. In *in vitro* studies, neither migration nor activation of CD40-B cells was inhibited by these immunosuppressive factors, nor did they influence the ability of CD40-B cells to induce proliferation of CD4+ or CD8+ T cells (216). This may in part explain some observations that tumor-infiltrating B cells have been associated with a better outcome (9–11), as they may provide better APC function in an immunosuppressive tumor microenvironment.

Kamphorst et al., have reported that if an antigen is processed after phagocytosis, conventional DCs are best suited for antigen presentation (128). They also demonstrated that B cells and DCs have similar efficacies if the antigen is acquired after receptor mediated endocytosis, but not if antigen enters by bulk phase endocytosis. These authors conclude that DC can process and present antigen similarly, irrespective of the antigen entry mechanism, whereas in the case of non-DC APCs, the entry mechanism can have a profound effect on antigen presentation (128). While in this report the authors studied antigen in protein form, antigen presentation by other antigen forms, for example encoded by DNA or RNA, could be different. In a study by Colluru et al., it was demonstrated that if DNA plasmid was passively delivered to APC, it was taken up by B cells by pinocytosis and the encoded antigen could be transcribed. On the other hand, other professional APC subtypes (DCs and macrophages) took up plasmid DNA by phagocytosis and failed to transcribe the encoded antigen (111). In a recent study of an mRNA-based vaccine and its effects on APC, Liang et al., demonstrated that multiple APC subsets could translate the vaccine mRNA *in vivo*. They showed that monocytes and DCs at the site of injection and in draining lymph nodes were mostly involved in translation of an mRNA vaccine. They also reported that B cells in draining lymph nodes could translate the mRNA vaccine. This was true for both intramuscular and intradermal injection routes, demonstrating that most professional APC subtypes can process RNA into antigen *in vivo* (217). In this study, the authors reported that DCs and monocytes preferentially presented the mRNA vaccine, relative to B cells, as there was an abundance of circulating DCs and monocytes at the site of injection.

In contrast to protein or nucleic acid sources of antigen, when presentation capabilities of different APC subsets were compared by Kamphorst et al. using peptide-loaded APCs, they reported that there were no significant differences between the APC subsets (128). Rosalia et al., have also shown that B cells and dendritic cells can present small synthetic peptide antigen to generate a similar T-cell response, as demonstrated by their ability to initiate proliferation of antigen-specific T cells (129). They also showed that if long synthetic peptides are delivered to APCs, which require subsequent processing by proteasomes and TAP-mediated MHC loading, then DCs are superior APCs, compared to B cells or macrophages (129). As described earlier, it remains possible that there could be other differences in the phenotype, or effector or memory function of T cells activated by different APC types, although this has not been extensively studied.

## 8 Use of B cells in immunotherapy

Various groups have studied ways of harnessing B cell APCs to improve immunotherapy (218). Our group has shown that B cells can serve as primary APCs in the context of DNA vaccines (111). Other groups have looked at ways of enhancing the APC function of human and murine B cells *via ex-vivo* stimulation with CD40, IL-4, or IFN-γ. These agents have been shown to increase MHC I and II expression as well as CD80/86 expression on B cells. The activated B cells were able to stimulate CD4+ and CD8+ T cells leading to increased proliferation in an antigen-specific manner (204, 206, 219, 220). When activated B cells were adoptively transferred into tumor-bearing mice, they caused superior control of tumor growth. Lee-Chang et al., showed that activated B cells that are adoptively transferred also produce tumor-specific antibodies that contribute to the anti-tumor response (220). These studies highlight the potential value of B cell APCs as therapeutic agents and provide a compelling reason why their function as APCs needs to be better understood.

DCs have been extensively studied as a cellular immunotherapy approach, typically by loading DC with protein/peptide/nucleic acid antigen as a vaccine. In contrast, there have been relatively few studies exploring B cells as APC vaccines. However, B cells may have clear advantages as APC cellular immunotherapies. As described above, CD40-B cells are technically easy to generate in large numbers, tolerate cryopreservation, and thus could potentially be used at a markedly reduced cost when compared to DC (221). One study tested the efficacy of CD40L- and IL-2-expressing autologous CLL B cells. The authors enrolled nine CLL patients out of which three patients showed >50% reduction in the size of affected lymph nodes and produced leukemia specific immunoglobulins (222). They also observed increased numbers of Treg cells before, during and after the treatment of these patients, suggesting that their presence may be the reason behind the transient response they observed and that their removal could be the key to augmenting and prolonging responses. Another study tested B cells as a cancer vaccine, using CD40-activated B cells in combination with chemotherapy in dogs with non-Hodgkin’s lymphoma. The authors observed improved second clinical remission and survival following this combination treatment (223). Two other clinical studies in human patients utilized allogeneic B cells from healthy donors fused with autologous tumor cells. In a trial of renal cell carcinoma patients that the received B cell-based vaccines, two complete and two partial remissions were observed out of 11 total patients enrolled (224). In a trial of metastatic melanoma patients, one complete remission, one partial remission and five patients with stable disease were reported out of 16 patients enrolled (225). While these were small studies, clearly the increased interest in B cells as vaccines or vaccine adjuvants will lead to further development over the next decade (226).

As previously discussed, the presence of TLS in solid tumors has been associated with favorable outcome, and the presence of B cells in these TLS has been of specific interest. In a study of melanoma patients, a correlation between increased survival and the presence of B cells along with activated (CD25+ or OX40+) T cells was demonstrated (227). Others have also demonstrated a correlation between increased overall survival in cancer patients and the presence of TIL-Bs along with CD8 T cells (228–231). These observations imply that the presence of B cells may be prognostic, and suggest that these B cells may be specific for tumor-associated antigens. It is also likely that TLS-residing B cells can capture tumor associated antigens and present to T cells. Treatment with B cell APCs might therefore prove to be beneficial. Most B cell-based treatment strategies have only been tested in early phase clinical trials with the primary goal of assessing their safety. However, it is evident from the recent *in vitro* and murine *in vivo* studies that CD40-activated B cell APCs function as antigen presenting cells to activate antigen specific CD4 and CD8 T cells with effector function (34). This implies that when CD40-activated B cells APCs are used as immunotherapy in cancer patients, they may perform an APC function and activate antigen specific T cells for cytotoxic functions. It is currently unknown whether antigen-pulsed B cells will be lysed by the T cells upon adoptive transfer, but this seems unlikely. It was shown by Watchmaker et al. that CD8 T cells release TNF-α before the cytotoxic granules (granzyme B and perforin), and this induces expression of PI-9 (granzyme B inhibitor) in DCs that protects them from CTL-mediated killing (232). Similar mechanism may exist for protection of B cells from CD8 CTL cytolysis to maintain an effective adaptive immune response upon vaccination.

Antigen-pulsed APCs are a direct way of delivering a vaccine antigen and eliciting an antigen-specific T cell based immune response that can have anti-tumor cytotoxic effects. Consequently, antigen-pulsed B cell APCs may be of significant interest as anti-cancer vaccines. However, there has been little study of this approach relative to studies evaluating antigen-pulsed DCs. Isolation, expansion, activation and then antigen pulsing autologous B cells should be far simpler than comparable studies using DC. As previously discussed, activation of B cells can be achieved by several agents, of which CD40 has been utilized by most groups. However, it has been demonstrated that when LPS-matured B cells are used for priming antigen-specific CD8 T cells, the resulting T cells are anergic in nature (233). This implies that the choice of activation agent for maturing B cells might have implications on the resulting phenotype of T cells. This requires further investigation. Moreover, B cell-mediated T-cell priming can result in distinct T cell phenotypes compared to priming through DCs; previous studies have demonstrated that T cells primed by DC or B cells may express different checkpoint receptor molecules (234). This further implies that use of B cell APCs as cancer vaccines might best be combined with checkpoint blockade therapy, and the choice of checkpoint blockade may be different from that used with DC-based vaccines.”

## 9 Summary

While the general role of B cells in tumor immunity has been extensively investigated, there are still gaps in knowledge that restrict our understanding of B cells as APCs. This has become increasingly important given that several studies have demonstrated that the presence of tumor-infiltrating B cells results in better prognosis, and these B cells can function as APCs. Several studies have demonstrated that stimulated B cells can elicit a similar, or in some cases a better, T cell-mediated anti-tumor response than other APC subsets. External stimuli such as TLR ligands, cytokines and other costimulatory molecules (such as CD40/CD40L and BAFF) have been evaluated for the ability to augment the antigen presentation capabilities of B cells. Some groups have attempted to use B cells as cellular vaccines, with early limited success. It is evident that a deeper understanding of B cell biology is necessary to more effectively harness the potential of B cells for cancer immunotherapy.

## Author contributions

Writing, IR and DM. Review and editing, DJ, JM, AM, HP, IR, and DM. Funding acquisition, DM. All authors contributed to the article and approved the submitted version.

## Funding

This research was funded by the National Institutes of Health R01 CA219154 and the Department of Defense Prostate Cancer Research Program (W81XWH-17-1-0247). JM is supported by NRSA award T32 CA009135.

## Acknowledgments

We would like to thank Christopher D. Zahm for his help during literature review for this manuscript.

## Conflict of interest

DM has ownership interest, has received research support, and serves as consultant to Madison Vaccines, Inc., which has licensed intellectual property related to this content.

The remaining authors declare that the research was conducted in the absence of any commercial or financial relationships that could be construed as a potential conflict of interest.

## Publisher’s note

All claims expressed in this article are solely those of the authors and do not necessarily represent those of their affiliated organizations, or those of the publisher, the editors and the reviewers. Any product that may be evaluated in this article, or claim that may be made by its manufacturer, is not guaranteed or endorsed by the publisher.

## Abbreviations

APC, Antigen-presenting cells; BAFF, B cell-activating factor; BCR, B cell receptor; Breg, B regulatory cells; Btk,; Bruton’s tyrosine kinase; CD40-B cells, CD40-activated B cells; DC, Dendritic cells; HSC, Hematopoietic stem cells; ICOS, Inducible co-stimulator; ICOSL, Inducible co-stimulator ligand; iNKT, invariant Natural killer T cells; MHC, Major histocompatibility complex; ODN, Oligodeoxynucleotides; PAMP, Pathogen Associated Molecular Patterns; SCID, Severe Combined Immunodeficiency; TIL, Tumor-infiltrating lymphocytes; TIL-B, Tumor-infiltrating B cells; TLR, Toll-like receptors; TLS, Tertiary lymphoid structures; Treg, T regulatory cells.
